# Trophoblast Cell Fusion and Differentiation Are Mediated by Both the Protein Kinase C and A Pathways

**DOI:** 10.1371/journal.pone.0081003

**Published:** 2013-11-13

**Authors:** Waka Omata, William E. Ackerman, Dale D. Vandre, John M. Robinson

**Affiliations:** 1 Department of Physiology and Cell Biology, The Ohio State University, Columbus, Ohio, United States of America; 2 Department of Obstetrics and Gynecology, The Ohio State University, Columbus, Ohio, United States of America; Fudan University, China

## Abstract

The syncytiotrophoblast of the human placenta is an epithelial barrier that interacts with maternal blood and is a key for the transfer of nutrients and other solutes to the developing fetus. The syncytiotrophoblast is a true syncytium and fusion of progenitor cytotrophoblasts is the cardinal event leading to the formation of this layer. BeWo cells are often used as a surrogate for cytotrophoblasts, since they can be induced to fuse, and then express certain differentiation markers associated with trophoblast syncytialization. Dysferlin, a syncytiotrophoblast membrane repair protein, is up-regulated in BeWo cells induced to fuse by treatment with forskolin; this fusion is thought to occur through cAMP/protein kinase A-dependent mechanisms. We hypothesized that dysferlin may also be up-regulated in response to fusion through other pathways. Here, we show that BeWo cells can also be induced to fuse by treatment with an activator of protein kinase C, and that this fusion is accompanied by increased expression of dysferlin. Moreover, a dramatic synergistic increase in dysferlin expression is observed when both the protein kinase A and protein kinase C pathways are activated in BeWo cells. This synergy in fusion is also accompanied by dramatic increases in mRNA for the placental fusion proteins syncytin 1, syncytin 2, as well as dysferlin. Dysferlin, however, was shown to be dispensable for stimulus-induced BeWo cell syncytialization, since dysferlin knockdown lines fused to the same extent as control cells. The classical trophoblast differentiation marker human chorionic gonadotropin was also monitored and changes in the expression closely parallel that of dysferlin in all of the experimental conditions employed. Thus different biochemical markers of trophoblast fusion behave in concert supporting the hypothesis that activation of both protein kinase C and A pathways lead to trophoblastic differentiation.

## Introduction

Cell-cell fusion is the cardinal event in the formation of multinucleated syncytia and is part of the normal biology of skeletal muscle, osteoclasts, and the syncytiotrophoblast (STB) layer of the human placenta. The placenta plays critical roles in many physiological functions of pregnancy including exchange of nutrients, ions, water, respiratory gases, hormones, vitamins, and other molecules necessary for fetal metabolism and development. Since the STB forms the interface with maternal blood, it is a key component in these processes. The STB also produces hormones necessary for the maintenance of pregnancy and plays a role in protecting the fetus from the maternal immune system. The STB is derived from and maintained by precursor cells, the mononuclear cytotrophoblasts (CTB). The CTBs fuse with the basal surface of the STB, a process important for placental growth and maintenance throughout pregnancy [1].

Dysferlin (DYSF) is a 230 kDa transmembrane protein related to *Caenorhabditis elegans* sperm vesicle-fusion protein, *Fer*-1 [2,3]. Dysferlin contains at least six C2 domains [4,5]. C2 domains are capable of binding phospholipids and proteins and the C2A domain of DYSF binds phospholipids in the presence of calcium [6,7]. Mutations in DYSF have been associated with limb girdle muscular dystrophy type 2B and Miyoshi myopathy [4,8-10]. Studies with isolated murine muscle fibers provide evidence that DYSF functions in repair of damaged sarcolemma in a calcium-dependent manner [11,12].

From a proteomics screen of the apical plasma membrane of the STB and other studies, we found that the protein DYSF was concentrated in the apical plasma membrane of the STB, but was not detected in CTBs [13-15]. We also showed that primary trophoblasts isolated from term placenta express DYSF following spontaneous fusion in culture [13]. Additionally, we showed that BeWo cells, a cell line often used as a surrogate for cytotrophoblasts, express DYSF following forskolin (FK)-induced cell fusion [15]. Thus, the expression of DYSF can be considered as another biochemical marker of trophoblast differentiation in the same fashion as the expression of other proteins such as syncytin-1, syncytin-2, and human chorionic gonadotropin (hCG), which are commonly accepted as STB differentiation markers. Further, the expression patterns of DYSF in BeWo cells, both before and after cell fusion, recapitulate the *in vivo* expression in the CTB and STB respectively, reiterating the usefulness of BeWo culture model as a surrogate system for studying trophoblast differentiation. 

It has been clearly established that elevation of intracellular cAMP through stimulation with forskolin or bromo-cAMP induces cell fusion and differentiation in BeWo cells [16]. Presumably, elevated cAMP acts upon cAMP-dependent protein kinase A (PKA) to induce changes associated with BeWo differentiation. Indeed, forskolin and bromo-cAMP have been the most commonly used stimulatory reagents used to study differentiation of BeWo cells. However, it has also been reported that 4β phorbol 12-myristate 13-acetate (PMA) leads to the production of the hormone hCG in BeWo cells [17]; hCG production is a classical biochemical marker of trophoblast differentiation. In addition, there are a limited number of reports using other trophoblast cell lines that further suggest protein kinase C (PKC) activation may also be capable of inducing properties of differentiation in trophoblasts [17,18]. We therefore hypothesized that PMA-treatment of BeWo cells would induce cell fusion and increase expression of DYSF and other markers of trophoblast differentiation such as syncytin-1, syncytin-2, and βhCG. In addition to demonstrating that PMA-treatment alone was capable of inducing trophoblast differentiation, we also showed that combined stimulation of both the PKA- and PKC-dependent pathways amplified, synergistically, the differentiation process in BeWo cells, inducing a temporally more rapid cell fusion as well as higher expression of fusion markers than either stimulatory agent when used alone.

## Materials and Methods

### Antibodies and chemicals

 A mouse monoclonal antibody to DYSF (Ham1) was purchased from Vector Laboratories (Burlingame, CA). A rabbit monoclonal antibody to E-cadherin (ab40772) was obtained from Abcam (Cambridge, MA). Mouse anti-glyceraldehyde 3-phosphate dehydrogenase (GAPDH) was from Covance

(Princeton, NJ). A mouse anti-β-human chorionic gonadotropin was obtained from Biodesign International (Saco, ME). Phospho-PKC (pan) β II (Ser 660) and phospho-PKC δ(Ser 643) antibodies were from Cell Signaling (Danvers, MA). Fluorochrome-labeled secondary antibodies, goat anti-mouse Alexa 594 and goat anti-rabbit Alexa 488, were from Molecular Probes/Invitrogen (Eugene, OR). Horseradish peroxidase-labeled goat IgG or donkey IgG were from Jackson ImmunoResearch (West Grove, PA). 4β phorbol 12-myristate 13-acetate (PMA) and forskolin (FK) were purchased from Sigma-Aldrich (St. Louis, MO). 4α phorbol 12-myristate 13-acetate (4α PMA) was from LC Laboratories (Woburn, MA). The protein kinase C inhibitor, bisindolylmaleimide I (Bis I), was obtained from Calbiochem/Merck Biosciences (Darmstadt, Germany). Protease inhibitor cocktail was purchased from Sigma-Aldrich. The BCA protein assay and Supersignal pico chemiluminescent kits were from Thermo Scientific (Rockford. IL). The βhCG ELISA kit was from DRG International Inc. (Springfield, NJ). 

### Cell culture

 The choriocarcinoma trophoblastic BeWo cell line was obtained from the American Type Culture Collection (Manassas, VA). The cells were maintained in F12/DMEM (1:1) medium (Invitrogen, Grand Island, NY) supplemented with 10% fetal bovine serum and penicillin/streptomycin (Sigma-Aldrich). The cells were grown at 37°C in a humidified atmosphere containing 5% CO_2_. To induce BeWo cell differentiation, cells were treated with 20 µM FK, various concentrations of PMA, or combined FK and PMA. The medium was exchanged with freshly prepared medium with or without stimulatory agents every 24 h. Control experiments lacked stimulatory agents but contained the solvent dimethyl sulfoxide (DMSO) added at the same concentration as that used with the stimulatory agents. 

### Immunoblotting

Control BeWo cells and cells treated with PMA, FK, or the combination of PMA and FK for 24, 48, and 72 h were collected with trypsin/EDTA (Sigma-Aldrich) and subsequently washed in PBS. Cell pellets were rapidly frozen in liquid nitrogen and stored at -80°C until used. The frozen pellets were resuspended in hot 2% SDS/PBS and boiled for 10 min. For phospho-PKC experiments, serum-starved cells (overnight) were treated with 100 nM PMA or 4α PMA for 0, 15, 30, 60, and 120 min. Cells were washed in cold PBS, subsequently lysed in 500 µl of cold homogenization buffer [20 mM Tris/HCl (pH 7.4),150 mM NaCl,1 mM EDTA,1 mM EGTA,1% Triton-X, 50 mM NaF,1 mM β-glycerophosphate, 1 mM Na_3_VO_4_, 2.5 mM sodium pyrophosphate,1 mM DTT, protease inhibitor cocktail]. After homogenization by passage through a 21G syringe on ice, cell lysates were centrifugated at 12,000 x *g* for 10 min at 4°C, and the supernatants were collected for immunoblotting. 

Soluble and particulate BeWo cell fractions were separated according to Rybin et al. [19]. Serum starved cells (overnight) were treated with 100 nM PMA for 60 min. Cells were washed in cold PBS, then placed in fractionation buffer [20 mM Tris/HCl (pH 7.4), 2 mM EDTA, 2 mM EGTA, 50 mM NaF, 1 mM β-glycerophosphate, 1 mM Na_3_VO_4_, 2.5 mM sodium pyrophosphate,1 mM DTT, and protease inhibitor cocktail]. Cells were scraped and transferred into ice cold tubes. Cells were lysed by sonication and centrifuged at 100,000 x *g* for 1 h. The supernatants were collected as the soluble fraction for immunoblotting. The resulting pellets were resuspended with fractionation buffer containing 1% Triton X-100 followed by centrifugation at 100,000 x *g* for 30 min at 4°C; these supernatants were collected as the particulate fraction for immunoblotting.

The protein concentration was determined with the Pierce BCA protein assay kit (Thermo Scientific, Rockford, IL). Equal concentrations of proteins were loaded and separated on SDS-polyacrylamide gels (6.0% for phospho-PKC; 7.5% for DYSF; 10.0% for βhCG) and then transferred to nitrocellulose membranes (Bio-Rad, Hercules, CA). The membrane was blocked in 5% milk, TBST [25 mM Tris/HCl (pH 7.4), 137 mM NaCl, 2.7 mM KCl and 0.05% (v/v) Tween 20] for 1 h at room temperature (RT), and then incubated overnight at 4°C with primary antibodies, anti-DYSF, anti-βhCG, anti-GAPDH, anti-phospho-PKC (pan) β II (Ser 660), or anti-phospho-PKC δ (Ser 643). After washing in TBST, membranes were incubated with species-appropriate HRP-labeled secondary antibodies (diluted 1:5,000) in milk/ TBST for 1 h RT. Following an extensive wash, the membranes were treated with Supersignal pico chemiluminescence system (Thermo Scientific) and exposed to X-ray film (Kodak, Rochester, NY).

### Immunofluorescence

 BeWo cells cultured on 22-mm square coverslips for 24, 48, or 72 h were fixed in freshly prepared 4% paraformaldehyde (PFA) in PBS for 1 h at RT. The cells were then washed in PBS and permeabilized with 0.5% sodium dodecyl sulfate in PBS for 5 min [20]. After washing in PBS for 1 h, non-specific protein-binding sites were blocked in 5% milk/PBS for 1 h. Cells were then incubated with primary antibodies overnight at 4°C. Cells were washed in PBS over 1 h and then incubated with secondary antibodies prior to indirect immunofluorescence microscopy. Alexa 488 conjugated anti-rabbit IgG and Alexa 594 conjugated anti-mouse IgG, each diluted 1:200 were incubated for 1 h at RT. Cells were then washed in PBS over 1 h and subsequently mounted on microscope slides in Prolong Gold anti-fade reagent containing DAPI (Invitrogen). Samples were observed with a Nikon Eclipse 90*i* microscope equipped with a motorized stage. Images were collected and analyzed with the Nikon Elements image analysis software package. Immunofluorescence controls did not receive the primary antibodies, but did receive secondary antibodies.

### Cell fusion assay

 Immunofluorescence microscopy was used to assess the levels of cell fusion. Random fields of view were obtained by use of the motorized stage in a blinded fashion. Images were then collected and a minimum of 1,000 nuclei were counted for each experimental condition. Cell fusion was assessed by monitoring the breakdown of the E-cadherin boundaries present in non-fused BeWo cells [21]. Additionally, we monitored the increase in DYSF expression in fused cells; thus, we had two indicators of cell fusion. In the fusion assay, the number of nuclei in syncytia (fused structures) and total number of nuclei were counted. A fusion index was calculated in the following manner: number of syncytial nuclei/total nuclei x 100. 

### Dysferlin knock down cell line: shRNA lentiviral transduction

 MISSION shRNA lentiviral transduction particles were obtained from Sigma-Aldrich (St. Louis, MO). The shRNA clones were designed and developed by The RNAi Consortium (Broad Institute, Cambridge, MA) using an algorithm to select and rank candidate hairpin sequences, each comprised of a 21 base stem and a 6 base loop, from Refseq transcripts reported from the NCBI gene database. The following hairpin shRNA sequence, cloned into the pLKO.1-puro vector, was used: 5’-CCG GCC GTG TAA TGT TTC AGG ATA ACT CGA GTT ATC CTG AAA CAT TAC ACG GTT TTT (#964). In control preparations, BeWo cells were incubated with MISSION Non-Target shRNA Control Transduction Particles (SHC002V, Sigma-Aldrich), as we have previously reported [22]. 

For single target knockdowns, BeWo cells were plated at 10^5^ cells/well in a 24-well tissue culture dish and incubated overnight. Transduction was carried out by adding concentrated lentiviral particles to the cells at a multiplicity of infection (MOI) of 1 in the presence of 8 µg/ml of hexadimethrine bromide. Following overnight incubation, the transduction medium was replaced with normal growth medium. On the second day following transduction, the cells were incubated in growth medium containing 8 µg/ml puromycin to select for puromycin-resistant colonies. We used these cells (BeWo 964) as DYSF knockdown BeWo cells.

### Measurement of human chorionic gonadotropin secretion

Cells were plated in 6-well plates at a density of 1.0 x 10^5^ cells/well and treated with PMA, FK, or the combination of PMA and FK for 24, 48, and 72 h. The culture medium was collected and stored at -80°C. The cells in each well were recovered and the cellular protein concentration was determined. The culture medium was thawed and centrifuged (500 x *g*); hCG in the medium was measured using a βhCG ELISA kit that recognizes both the intact protein and free βhCG. These data were normalized to cellular protein concentration. 

### Quantitative real-time PCR

Total RNA was isolated with NucleoSpin RNA ll (Clontech, Mountain View, CA) according to the manufacturer's protocol. Total RNA was quantified by spectrophotometry with a NanoDrop 2000 (Thermo Scientific, Waltham, MA), and 1 to 5 µg total RNA from each sample was reverse-transcribed to cDNA using RNA to cDNA EcoDry Premix (Oligo dT) (Clontech, Mountain View, CA). Quantitative RT-PCR was performed using an equal amount of cDNA per sample on a LightCycler480 II System (Roche, Indianapolis, IN) using primer-probe sets specific to DYSF, syncytin 1 (ERVW-1), syncytin 2 (ERVFRD-1), and large ribosomal protein (RPLPO) (Applied Biosystems, Carlsbad, CA) with the LightCycler 480 Probes Master Mix (Roche, Indianapolis, IN). PCR reaction was carried out as follows, preincubation at 95°C for 10 s and amplification at 95°C for 10 s, at 60°C for 30 s, at 72°C for 1s for 45 cycles and cooling at 40°C for 10 s. Results were analyzed with the LightCycler 480 Software.

### Statistical analysis

Values are given as mean ± standard deviation of at least three independent experiments. Prior to statistical analysis, the D’Agostino & Pearson omnibus normality test was applied to determine whether parametric or non-parametric statistical testing was appropriate in each case. Statistical analysis was performed using either the one-way ANOVA followed by Bonferroni’s multiple comparisons test or the Kruskal-Wallis followed by Dunn’s multiple comparison test. Comparisons between two groups were carried out with the 2-tailed student t-test for unpaired samples. All statistical tests were done using GraphPad Prism software (San Diego, CA) and are indicated in the text and figure legends.

## Results

### Activation of protein kinase C enhances cell fusion and differentiation in BeWo cells

**Figure 1:**
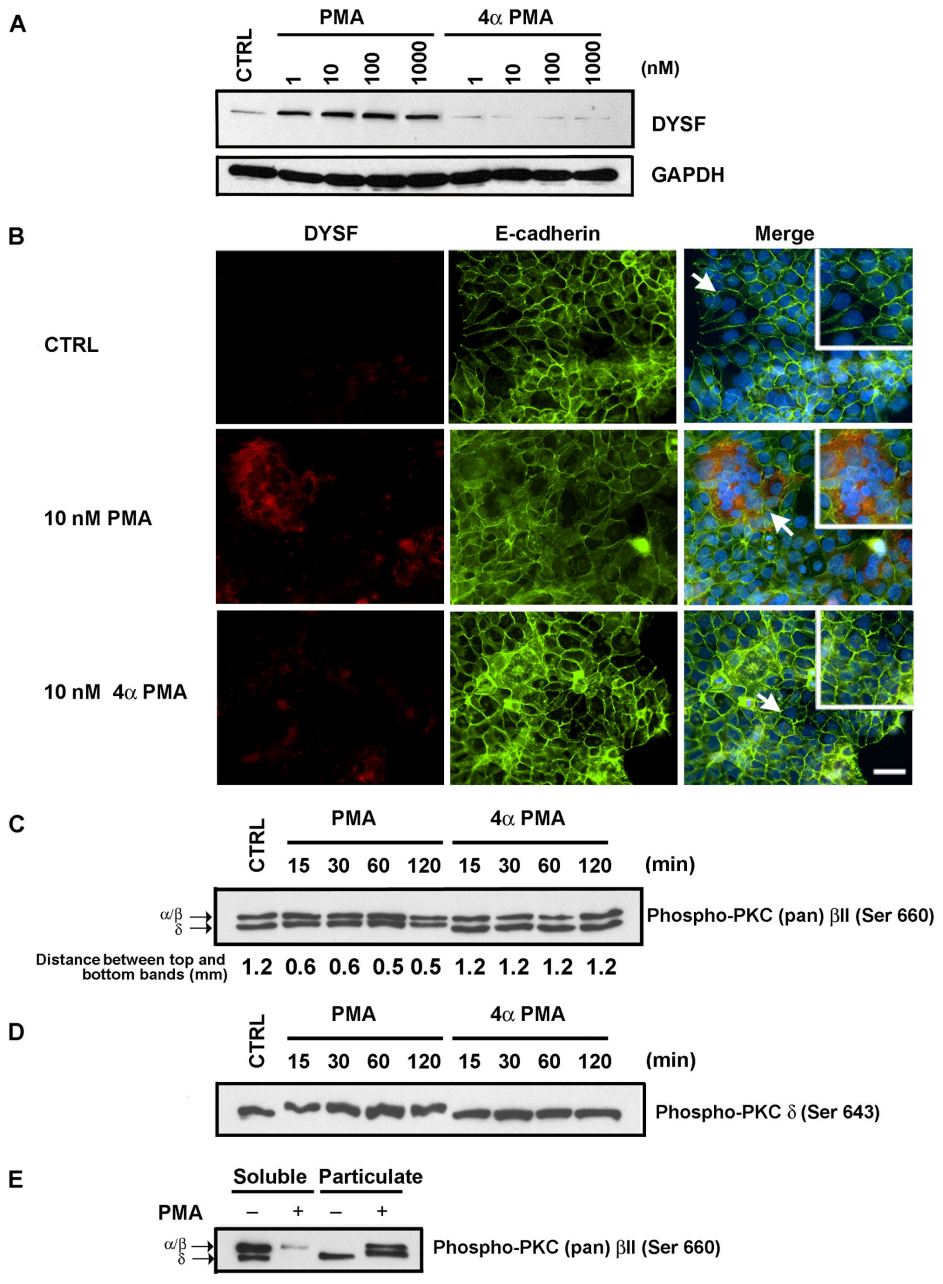
PMA induced cell fusion, DYSF expression, and activation of PKC in BeWo cells while 4αPMA was inactive. (A) BeWo cells were treated with 0.25% DMSO (solvent control, CTRL) or with PMA (1, 10, 100, 1000 nM) or 4αPMA (1, 10, 100, 1000 nM) for 72 h. Cell lysates were generated and immunoblots were probed with anti-DYSF. Each lane received equal amounts of protein and detection of GAPDH served as an additional loading control. Up-regulation of DYSF occurred with all concentrations of PMA tested. On the other hand, none of the concentrations of 4αPMA tested induced DYSF expression. (B) Immunofluorescence analysis of BeWo cells treated with 0.25% DMSO (controls), 10 nM PMA, or 10 nM 4αPMA for 72 h. The cells were then fixed and subsequently double-labeled for detection of DYSF (red) and E-cadherin (green). Nuclei were labeled with DAPI. While there can be a low level of spontaneous fusion in control cells (in our hands this ranges from about 4 to 9%), most cells are not fused and have at their borders intact E-cadherin labeling. Moreover, DYSF labeling was not detectable in non-fused BeWo cells. However, treatment of BeWo cells with 10 nM PMA for 72 h led to increased levels of cell fusion as indicated by the breakdown of E-cadherin labeling and the expression of DYSF in fused cells. When BeWo cells were treated with 10 nM 4αPMA for 72 h there was no detectable increase in cell fusion or DYSF expression. Arrows indicate areas enlarged and placed in insets. Bar = 50 µm. (C, D) Activation of PKC with PMA but not with 4αPMA. The electrophoretic mobility of BeWo cell PKC was monitored using a phospho-PKC (pan) β II (C) and phospho-PKC δ(Ser 643) (D) antibodies. (C) In control cells (DMSO treated), two bands were detected with this antibody indicating a basal level of PKC (pan) β II phosphorylation. Following treatment with PMA, there was a change in the mobility of the lower band over the time course of this experiment (15, 30, 60, and 120 min). This change in mobility was not observed following 4αPMA treatment. The distances between the top and bottom bands were measured on the original x-ray films; these distances are indicated in the measured distances (mm). Phospho-PKC (pan) antibody detects both α and β isoforms, thus the upper band was labeled α/β. (D) In control cells (DMSO treated), this antibody detected a basal level of PKC δ phosphorylation. Following treatment with PMA, there was a change in the mobility of this band over the time course of this experiment (15, 30, 60, and 120 min). This change in mobility was not observed following 4αPMA treatment. (E) Phospho-PKC (pan) β II was translocated from soluble to particulate fraction with PMA treatment. In control cells (DMSO treated), two bands were detected in soluble fraction and only lower band was in particulate fraction with phospho-PKC (pan) βII. Following treatment with 100 nM PMA for 1 h, phospho- PKC (pan) β II was translocated to particulate fraction and there was a change in the mobility of the lower band as described in Figure 1C. Results are representative of three independent experiments.

We first examined whether PMA induced DYSF expression in BeWo cells. After treatment with PMA for 72 h, we observed that PMA induced DYSF expression over a broad range of concentrations (1 to 1000 nM) (Figure 1A). Over that same range of concentrations, 4α PMA (the stereo isomer that does not activate PKC) failed to induce DYSF expression. We also examined cell-cell fusion in response to these agents. In control BeWo cells, there was a low level of spontaneous fusion similar to that reported by others [23], but most cells were mononuclear and did not exhibit DYSF induction (Figure 1B). 

 PMA, but not 4α PMA, induced cell fusion as assessed by loss of E-cadherin expression using immunofluorescence microscopy (Figure 1B). We previously reported that DYSF expression was induced in BeWo cells following forskolin mediated cell-cell fusion [15]. Using the phospho-PKC (pan) β II antibody (which detects endogenous levels of several phosphorylated PKC isoforms, including α, β and δ) we compared PMA and 4αPMA for their ability to activate PKC. The immunoblot assay detected two distinct PKC bands (~78kDa and 81kDa) in control cells, indicating a basal level of PKC phosphorylation. Following treatment with PMA, there was a shift in electrophoretic mobility of the lower band without an increase in labeling intensity, suggesting increased phosphorylation (Figure 1C) at a residue other than that detected with this antibody. This shift was evident by 15 min and persisted for at least 120 min. Treatment with 4 αPMA, the steroisomer that does not activate PKC, did not result in a mobility shift in the ~78 kDa band (Figure 1C). As the phospho-PKC (pan) β II antibody detects several PKC isoforms, we repeated the analysis using an antibody directed against PKC δ when phosphorylated at serine residue 643. Following treatment with PMA, but not 4αPMA, there was also a shift in electrophoretic mobility (but not an increase in labeling) of phospho-PKC δ (Figure 1D). To further confirm PKC activation, we investigated cellular localization (Figure 1E). Following treatment with PMA, phosphorylated PKC was translocated from the soluble fraction to a particulate (membrane-associated) fraction (Figure 1E). Translocation of PKC from the soluble to the particulate fraction is a hallmark of PKC activation. 

**Figure 2:**
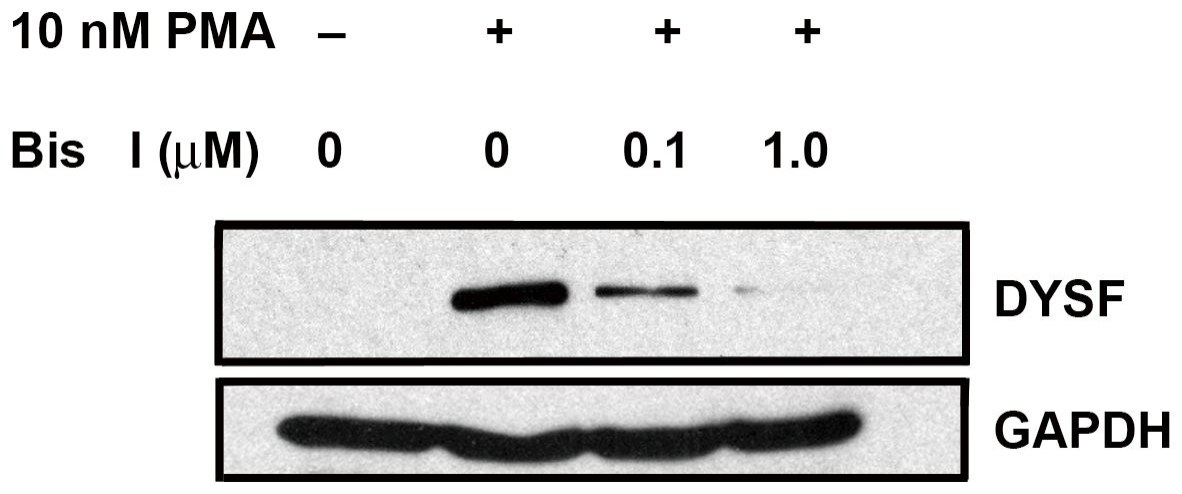
Bis I inhibited PMA-induced DYSF expression in a dose-dependent manner. Cells were treated with 0.25% DMSO (CTRL), 10 nM PMA, or 10 nM PMA plus 0.1 or 1.0 µM Bis I for 72 h. Cell lysates were generated and immunoblots were probed with anti-DYSF. Each lane received an equal concentration of protein and detection of GAPDH served as an additional loading control. Results are representative of three independent experiments.

**Figure 3:**
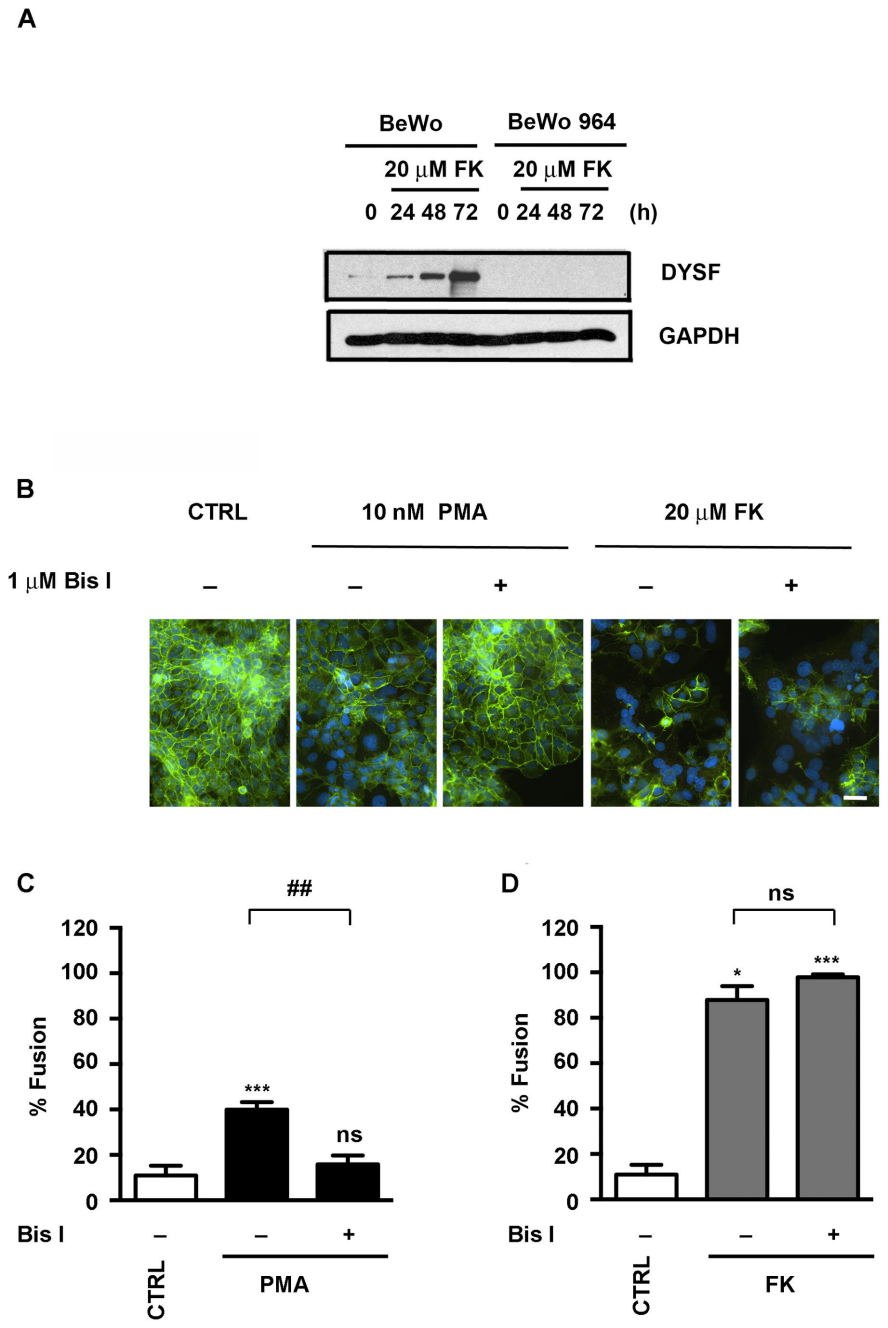
The PKC inhibitor Bis I inhibited cell fusion induced by PMA but not FK-induced cell fusion in DYSF knock down BeWo cells. (A) While DYSF expression is increased in a time-dependent manner following treatment with FK in the parental BeWo cells, barely detectable DYSF expression was found in BeWo 964 DYSF-knockdown cells following FK treatment. (B) Immunofluorescence assay shows that Bis I inhibits PMA-induced cell fusion but not FK-induced fusion. Cells were incubated as indicated for 72 h prior to fixation and subsequent labeling of E-cadherin (green) and nuclei (blue). Bar = 50 µm. (C) The immunofluorescence assays were quantified and reported as percentage of nuclei in syncytia (% fusion) following PMA-treatment with or without Bis I. (D) The immunofluorescence assays were quantified and reported as percentage of nuclei in syncytia (% fusion) following FK-treatment with or without Bis I. The results are the mean ± SD (n = 3). *P < 0.05; ***P < 0.001 (*vs*. control), ^##^P < 0.01 (PMA *vs*. PMA + Bis I), ns: not significant (PMA + Bis I *vs*. control; FK *vs*. FK + Bis I) by Kruskal-Wallis/Dunn.

To confirm that PMA induced cell fusion and DYSF expression in a PKC-dependent manner, we examined the effect of the PKC inhibitor Bis I on these events. In immunoblot assays, we found that Bis I inhibited PMA-induced DYSF expression in a concentration-dependent manner following 72 h of treatment (Figure 2). Simultaneously, treatment with Bis I also blocked PKC induced cell-cell fusion in BeWo (see below, Figure 3). These results indicate that PMA and FK induce fusion via separate signaling pathways, as demonstrated by the sensitivity of PMA-induced differentiation to Bis I, DYSF was up-regulated in fused cells in response to either treatment. 

### Expression of DYSF is not necessary for BeWo cell fusion in response to PMA or forskolin

 The DYSF knockdown cell line, BeWo 964, expresses little or no DYSF in response to FK stimulation (Figure 3A) and undetectable levels in response to PMA treatment (data not shown). As shown by the loss of immunofluorescence staining of E-cadherin following cell fusion, DYSF deficient BeWo 964 cells fused in response to either PMA or FK (Figure 3B). Moreover, PMA-induced cell fusion was inhibited by Bis I, but FK-induced fusion was unaffected by Bis I treatment (Figure 3B). The degree of BeWo 964 cell fusion was quantified under each of these treatment conditions. The level of PMA-induced fusion in BeWo 964 cells was elevated; treatment of the cells with Bis I reduced PMA-induced fusion to control levels (Figure 3C). The level of FK-induced fusion in BeWo 964 cells was elevated; treatment of the cells with Bis I did not inhibit FK-induced fusion (Figure 3D). Comparison of parental BeWo cells for fusion induced by PMA (41.4 ± 10.4%) or FK (92.1 ± 5.8%) at 72 h with BeWo 964 cells treated with PMA (40.0 ± 3.3%) or FK (87.9 ± 6.1%) at 72 h showed no significant differences as indicated by the student t-test. While DYSF expression is normally induced following cell fusion, the DYSF knockdown cells demonstrate that its expression is not required for the fusion process. 

### PMA amplifies FK-induced DYSF expression

**Figure 4:**
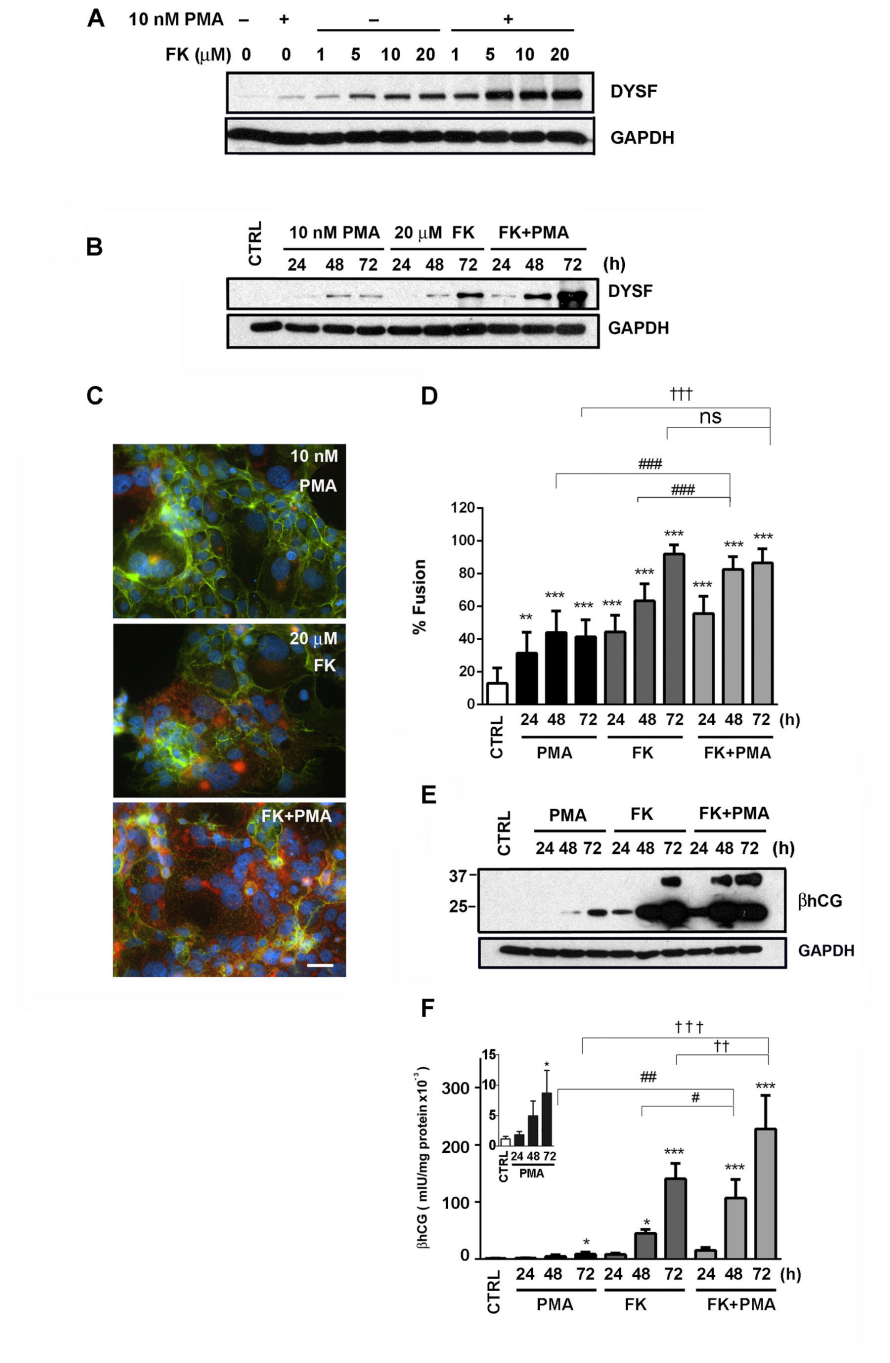
Foskolin-induced differentiation was augmented by PMA. (A) An immunoblot showing the dose-response for FK in the presence or absence of 10 nM PMA. Note that PMA augments the expression of DYSF at each concentration of FK tested. Each lane received an equal concentration of proteins and detection of GAPDH served as an additional loading control. Results are representative of three independent experiments. (B) The time course for DYSF expression in the presence of 10 nM PMA, 20 µM FK, or the combination of FK and PMA. Note that there was increased DYSF expression with the combination of FK and PMA at each time point tested when compared to PMA or FK alone. Also with the combination of FK and PMA, DYSF expression was evident by 24 h while it lagged behind with PMA or FK alone under these conditions. Results are representative of three independent experiments. (C) Immunofluorescence localization of DYSF (red) and E-cadherin (green) following 72 h treatment with 10 nM PMA, 20 µM FK, or a combination of FK and PMA; the nuclei were stained with DAPI (blue). Note the enhanced fluorescence signal for DYSF in the FK + PMA sample when compared to FK or PMA alone. Bar = 50 µm. (D) The immunofluorescence assays were quantified and reported as percentage of nuclei in syncytia (% fusion). Results are the mean ± SD (n = 3). **P < 0.01; ***P < 0.001 (vs. control), ^###^ P < 0.001 (48 h PMA *vs*.48 h FK+PMA; 48 h FK vs. 48 h FK+PMA), ^†††^P < 0.001 (72 h PMA *vs*. 72 h FK+PMA), ns: not significant (72 h FK *vs*.72 h FK + PMA) by one-way ANOVA/Bonferroni. (E) The time course for the expression of cell-associated βhCG protein in response to 10 nM PMA, 20 µM FK, or the combination of PMA and FK is shown. Control cells do not have detectable βhCG. While PMA does induce the expression of βhCG, it is at a modest level when compared to treatment with FK. The stimulation of BeWo cells with PMA and FK simultaneously induces higher levels of βhCG than FK alone; this is most evident at 24 h treatment. The immunoblot and immunofluorescence data in this figure are representative of at least three independent experiments. (F) The time course for βhCG secretion in response to 10 nM PMA, 20 µM FK, and a combination of PMA and FK is shown. Each treatment induced βhCG secretion with PMA + FK > FK > PMA. The results are the mean ± SD (n = 3). *P < 0.05; ***P < 0.001 (vs. control), ^#^P < 0.05; ^##^ P < 0.01 (48 h FK *vs*.48 h FK+PMA; 48 h PMA vs. 48 h FK+PMA), ^††^P < 0.01; ^†††^P < 0.001 (72 h FK *vs*. 72 h FK+PMA; 72 h PMA *vs*. 72 h FK+PMA) by one-way ANOVA/Bonferroni.

We used an immunoblot approach to determine whether PMA may synergize with FK to induce DYSF expression. Using a fixed concentration of PMA (10 nM), the expression of DYSF was enhanced at each concentration of FK tested (Figure 4A). We next carried out time-course experiments to assess DYSF expression following stimulation with PMA, FK, or PMA + FK. For each of these treatments, DYSF expression increased in a time-dependent manner (Figure 4B). While FK (20 µM) induced higher expression of DYSF than did PMA (10 nM), there was a dramatic increase in DYSF when PMA and FK were used in combination (Figure 4B). Moreover, when used in combination, DYSF expression was detected earlier than with PMA or FK alone. Using immunofluorescence microscopy, we found that high levels of cell fusion occurred in response to FK alone (20 µM) as well as PMA (10 nM) + FK (20 µM) as evidenced by decreased E-cadherin labeling (Figure 4C). While each treatment led to increased DYSF labeling in fused cells, the intensity of DYSF staining showed that PMA + FK > FK > PMA (Figure 4C). The immunofluorescence data were thus consistent with the immunoblot data. The labeling experiments were also used to quantify BeWo cell fusion under the different stimulatory conditions (Figure 4D). There was a time-dependent induction of fusion with each treatment; at 72 h, PMA was less effective (~42%) than either FK alone or PMA + FK (each ~90-95%). While FK alone and PMA + FK induced about the same level of fusion, there was clearly enhanced production of DYSF in the PMA + FK samples.

### PMA amplifies FK-induced human chorionic gonadotropin expression

Another marker of trophoblast differentiation, expression of βhCG, was monitored over time following stimulation of BeWo cells with PMA, FK, or FK + PMA. While most investigators measure hCG secreted into the culture medium, we also monitored cell-associated βhCG by immunoblotting. We found that control cells exhibited undetectable levels of βhCG (Figure 4E). Incubation with PMA led to modest levels of βhCG production that was not detectable until 48 h of treatment (Figure 4E). On the other hand, FK treatment led to a much more robust expression of cell-associated βhCG (Figure 4E). The combination of PMA and FK produced an even more robust signal than FK alone, which was most obvious following 24 and 48 h of incubation (Figure 4E). Next, we measured βhCG secretion by ELISA. The release of βhCG was increased by PMA or FK alone in a time dependent manner (Figure 4F). Secretion of βhCG was increased in a synergistic manner when PMA and FK were combined (Figure 4F), mirroring the immunoblotting results (Figure 4E). We next compared PMA and 4αPMA for their ability to induce production of the trophoblast differentiation marker βhCG. Cell-associated βhCG was detected at PMA concentrations of 10, 100, and, 1000 nM. On the other hand, 4αPMA did not induce βhCG production at any of these concentrations (Figure S1). We also tested of the ability of the PKC-inihibitor, Bis I for its effect in blocking PMA-induced production of βhCG. Treatment of BeWo cells with 0.1 µM or 1.0 µM Bis I led to a marked reduction in PMA-induced cell–associated βhCG (Figure S2).

### Real time qPCR analysis of DYSF and cell-fusion related genes

**Figure 5:**
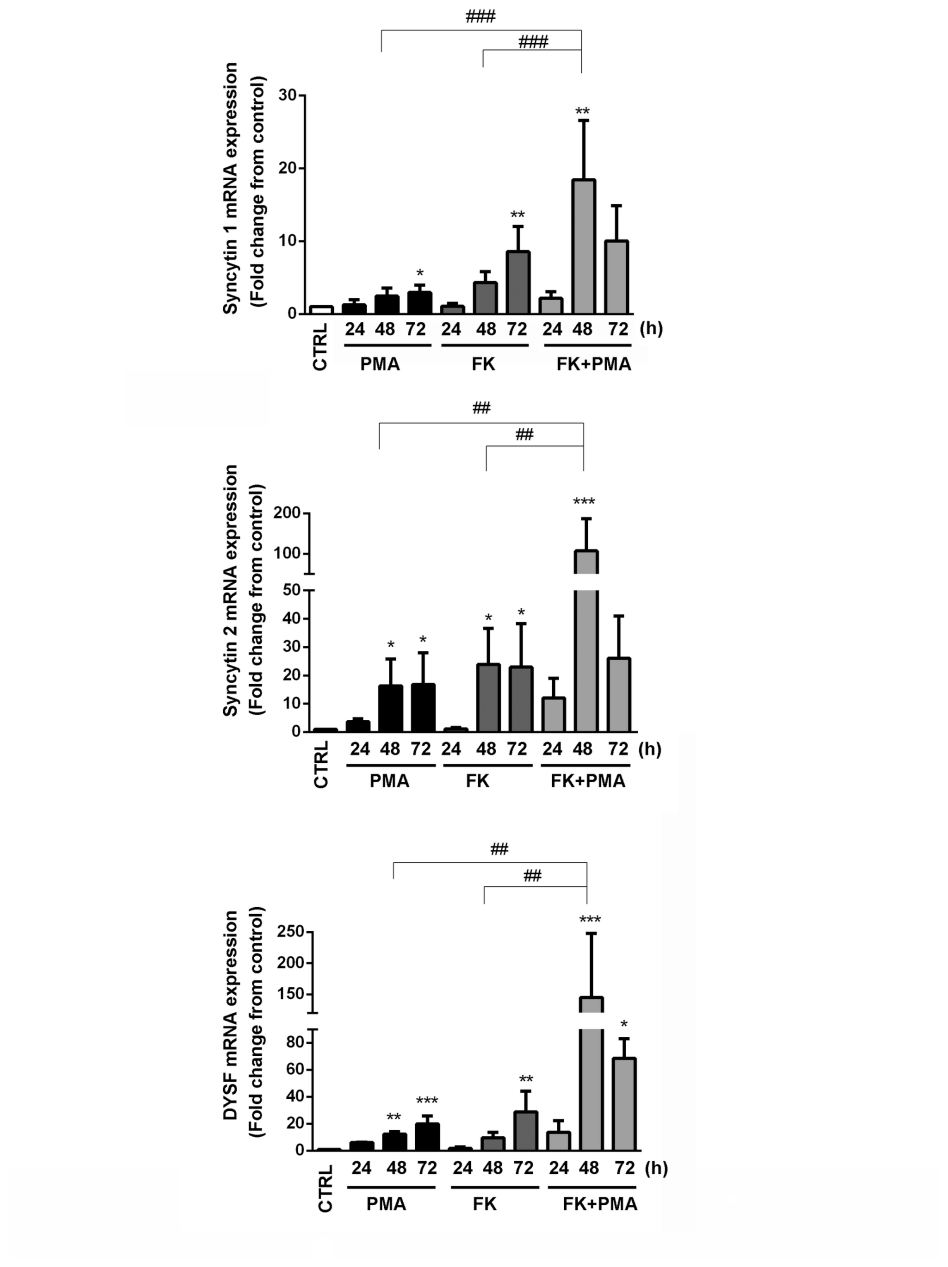
The time course for quantitative PCR analysis of mRNA expression for syncytin 1, syncytin 2, and DYSF in response to treatment of BeWo cells with 10 nM PMA, 20 µM FK, or a combination of FK+ PMA. All mRNA expression was normalized to RPLPO. Treatment of BeWo cells with PMA resulted in increased expression of mRNAs for syncytin 1, syncytin 2, and DYSF. However, these increases were not as high as those found with FK treatment. Simultaneous treatment of cells with PMA + FK led to dramatic increases in mRNAs for syncytin 2 and DYSF that peaked at 48 h of treatment. Increases in syncytin 1 mRNA was not as dramatic as for syncytin 2. Results are the mean ± SD (n = 3). *P < 0.05; **P < 0.01; ***P < 0.001 (vs. control), ^##^ P < 0.01; ^###^ P < 0.001 (vs. 48 h FK+PMA) by one-way ANOVA/Bonferroni.

Having observed cell fusion following treatment with PMA, FK, and PMA + FK, we asked whether the trophoblast fusion-proteins syncytin1 and syncytin 2 were up-regulated following these treatments. We utilized quantitative PCR to determine changes in the mRNA levels following each treatment. As a positive control we also monitored DYSF mRNA. We found that PMA and FK alone each led to a time-dependent increase in mRNAs for syncytins 1 and 2, as well as DYSF (Figure 5). Interestingly, the level of syncytin 2 mRNA induction was about twice that for syncytin 1. Even more striking was the up-regulation of syncytin 2 and DYSF mRNAs when PMA and FK were used in combination; there was a synergistic increase in these mRNAs that peaked at 48 h with at least a 100-fold increase over control samples. There was no synergistic increase in syncytin 1 mRNA under these same conditions. These data correlate with the observed changes in cell-cell fusion and DYSFand βhCG protein expression, and demonstrate that syncytin 2 and DYSF mRNA expression patterns respond similarly to the various differentiation stimuli.

## Discussion

Differentiation of CTBs into the STB is a process required for the development of a functional placenta [1]. CTB differentiation is characterized by both biochemical and morphological alterations, including up-regulation and expression of human chorionic gonadotropin and human placental lactogen, production of estrogens and progesterone, and fusion of cells resulting in syncytium formation [24]. While these changes appear to be coupled, it is not clear whether some of the biochemical processes can occur, at least in part, in the absence of cell fusion, and vice versa. Regardless, full CTB differentiation only occurs following cell fusion. We have previously shown that DYSF expression is tightly coupled with trophoblast cell fusion [13,15]. While the function of DYSF in the STB remains to be elucidated, it is likely to play a role in membrane repair, membrane maintenance, and/or vesicular transport as has been reported in other systems. For example, in endothelial cells it has been reported that DYSF mediates lysosome fusion to the plasma membrane [25] and trafficking of platelet endothelial cell adhesion molecule 1 to the plasma membrane [26]. Abnormal trafficking of insulin-like growth factor receptor has been noted in DYSF-null myoblasts [27]. Thus, it may be that DYSF is involved in multiple intracellular fusion events in differentiated trophoblasts.

The BeWo choriocarcinoma cell line has been used as a model system for studying the differentiation of trophoblasts, since it was observed that BeWo cells can be induced to fuse following treatment with compounds such as forskolin that induce elevated cAMP levels [16]. Differentiation of BeWo cells mediated by cAMP was further shown to be dependent upon activation of PKA [28]. In addition to forming a syncytium in the presence of forskolin, fused BeWo cells also express differentiation markers such as βhCG that are associated with STB function, and proteins such as DYSF that are coupled to membrane fusion. 

Induction of βhCG expression has also been reported in first trimester trophoblasts and BeWo cells following PMA treatment, which activates PKC signaling [29-31]. It has been shown that simultaneous activation of both PKA-and PKC-dependent pathways in trophoblast cell lines results in a synergistic increase in βhCG levels [17,18], but further markers of biochemical or morphological differentiation were not determined. Thus, we questioned whether treatment of BeWo cells with PMA alone, or in combination with FK, would lead to enhanced cell fusion and/or increased expression of DYSF and other markers of trophoblast differentiation such as the syncytins and βhCG.

 In the results presented here, we show that treatment of BeWo cells with PMA induces cell-cell fusion and DYSF up-regulation, albeit moderately in comparison to the effects of FK alone. The susceptibility of these effects to Bis I inhibition indicates dependence on PKC signaling, which is further supported by the lack of such responsiveness to equivalent concentrations of 4αPMA, an isomer of PMA that is unable to activate PKC [32].

 We used two complementary strategies to determine whether PMA activates PKC in BeWo cells. Using an antibody that recognized most of the PKC isoforms when phosphorylated at residues homologous to serine 660 of PKC β II, two distinct bands were detected by immunoblotting. The lower of these two bands displayed a mobility shift in cells treated with PMA, but not in control cells or those treated with 4αPMA. The lower band was subsequently identified as PKC δ with an antibody that recognizes this isoform when phosphorylated at serine 643. These results are consistent with a change in the electrophoretic mobility of PKC δ following PMA treatment and are similar to that reported in cardiomyocyte cultures [19]. A hallmark of PKC activation is the translocation of PKC isoforms from a predominately soluble localization to a predominately particulate localization following treatment with PMA [19,33]. BeWo cells with or without treatment with PMA were disrupted to generate soluble and particulate membrane associated fractions by centrifugation. In these experiments, there was a dramatic depletion of soluble phospho-PKC (pan) and a parallel increase in phospho-PKC (pan) in the particulate fraction following PMA treatment. Taken together, these data provide strong evidence for the activation of PKC by PMA in BeWo cells.

These results provided an association between BeWo cell fusion and DYSF expression. However, these data did not distinguish whether one of these events (fusion vs. DYSF expression) was proximal. To approach this question, we used DYSF-knockdown BeWo cells in conjunction with Bis I and stimulation with either PMA or FK. Both PMA and FK induced fusion of the DYSF-knockdown cells, although FK induced twice the level of fusion as PMA as determined by the cell-fusion assay. The PKC inhibitor significantly blocked PMA-induced cell fusion but did not inhibit FK induced fusion. These data support the contention that Bis I-sensitive and Bis I-insensitive fusion pathways are present in BeWo cells. Additionally, these results show that DYSF expression is not required for BeWo cell fusion, since the DYSF knockdown cells do not express detectable levels of DYSF. Thus DYSF expression is normally up-regulated in response to trophoblast cell fusion, and unlike syncytin, DYSF expression does not appear to be necessary for initiation or completion of the fusion process. Thus, these data refute the idea that DYSF is necessary for BeWo cell fusion. Additionally, we confirmed that treatment of BeWo cells with PMA induces expression of βhCG at the protein level and that it was secreted from the cells (Figure 4F, inset), but this expression is also modest when compared to FK treatment. Thus, it appears that there are two distinct fusion pathways available to BeWo cells, one induced by PMA and the other by FK and proceeding *via* the PKC and PKA signaling pathways respectively. 

We next asked if treating BeWo cells with PMA and FK simultaneously would lead to a greater response than when these reagents were used individually. When PMA was held at a constant dose (10 nM) and the FK dose was varied (1, 5, 10, 20 µM) there was a dramatic increase in DYSF expression; this occurred at each FK concentration compared to FK alone. In a time course experiment comparing stimulation with PMA (10 nM), FK (20 µM), or PMA + FK, it was confirmed that the combination of stimuli dramatically increased DYSF expression; this occurred at each time point tested. When PMA and FK were used in combination, DYSF was readily detectable within 24 h of stimulation; thus, the combination of PMA and FK induced more rapid expression of DYSF than PMA or FK alone. Comparison of PMA, FK, and PMA + FK in immunofluorescence experiments reinforced the immunoblot data. There was a more intense fluorescent signal for DYSF localization with PMA + FK in fused cells than with PMA or FK stimulation alone. Further, the expression of cell-associated βhCG as well as secreted βhCG was also significantly augmented when BeWo cells were simultaneously treated with PMA + FK.

Having observed cell fusion following treatment with PMA, FK, and PMA + FK we asked whether the trophoblast fusion-proteins syncytin 1 and syncytin 2 were upregulated following these treatments. Since we did not have access to useful antibodies to syncytin 1 or syncytin 2, we approached this question using quantitative PCR methods. As a positive control we also monitored DYSF mRNA. We found that PMA and FK alone led to a time-dependent increase in mRNAs for syncytin 1 and 2, as well as DYSF. Interestingly, the level of syncytin 2 mRNA production was about twice that of syncytin 1 in response to treatment with either of these fusogenic agents alone. Even more striking was the up-regulation of syncytin 2 and DYSF mRNAs when both PMA and FK were used to stimulate the cells; there was a synergistic increase in these mRNAs that peaked at 48 h. There was not a corresponding synergistic increase in syncytin 1 mRNA under these conditions, rather the combination of PMA and FK resulted in producing a slightly more than an additive response. These data, at the mRNA level, support our observation that there was a dramatic increase in DYSF at the protein level following simultaneous treatment of BeWo cell with PMA and FK. Additionally, these data provide new information related to the increases in both syncytin 1 and syncytin 2 mRNA following PMA treatment of BeWo cells. Moreover the synergistic increase in syncytin 2 mRNA that accompanies stimulation with the combination of PMA and FK is remarkable and unexpected. Further investigation will be required to fully decipher the underlying pathways responsible for this synergistic response. 

## Supporting Information

Figure S1 **PMA induced the expression of cell-associated βhCG protein in BeWo cells while 4αPMA failed to induce βhCG.** BeWo cells were treated with 0.25% DMSO for controls (CTRL) or with PMA (1, 10, 100, 1000 nM) or 4αPMA (1, 10, 100, 1000 nM) for 72 h. Cell lysates were generated and immunoblots were probed with anti-βhCG. Each lane received equal amounts of protein and detection of GAPDH served as an additional loading control. Up-regulation of βhCG was observed over 10 nM PMA, whereas 4αPMA did not induce βhCG production at any of the concentrations tested.
(TIF)

Figure S2 **Bis I inhibited PMA-induced βhCG protein expression.** Cells were treated with 0.25% DMSO (CTRL), 10 nM PMA, or 10 nM PMA plus 0.1 or 1.0 µM Bis I for 72 h. Cell lysates were generated and immunoblots were probed with anti-βhCG. Each lane received an equal concentration of protein and detection of GAPDH served as an additional loading control.
(TIF)
