# Supplementary figures and images for: Trophoblast Cell Fusion and Differentiation Are Mediated by Both the Protein Kinase C and A Pathways
Source: PLoS One. 2013 Nov 13;8(11):e81003. doi: 10.1371/journal.pone.0081003 (PMC3827470; doi:10.1371/journal.pone.0081003)

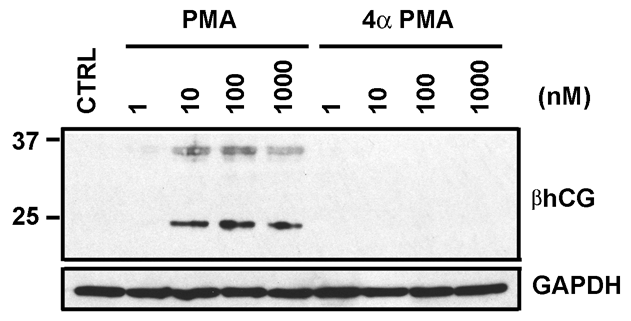

Supplement: Figure S1 — PMA induced the expression of cell-associated βhCG protein in BeWo cells while 4αPMA failed to induce βhCG. BeWo cells were treated with 0.25% DMSO for controls (CTRL) or with PMA (1, 10, 100, 1000 nM) or 4αPMA (1, 10, 100, 1000 nM) for 72 h. Cell lysates were generated and immunoblots were probed with anti-βhCG. Each lane received equal amounts of protein and detection of GAPDH served as an additional loading control. Up-regulation of βhCG was observed over 10 nM PMA, whereas 4αPMA did not induce βhCG production at any of the concentrations tested. (TIF) [file pone.0081003.s001.tif]

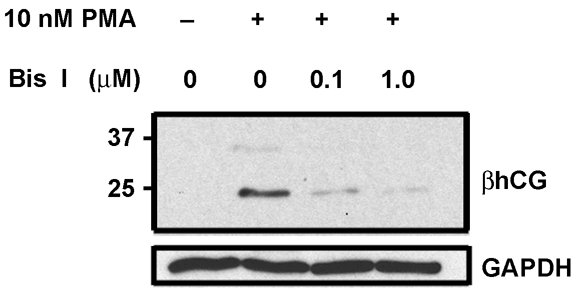

Supplement: Figure S2 — Bis I inhibited PMA-induced βhCG protein expression. Cells were treated with 0.25% DMSO (CTRL), 10 nM PMA, or 10 nM PMA plus 0.1 or 1.0 µM Bis I for 72 h. Cell lysates were generated and immunoblots were probed with anti-βhCG. Each lane received an equal concentration of protein and detection of GAPDH served as an additional loading control. (TIF) [file pone.0081003.s002.tif]
